# Garden cress gum and maltodextrin as microencapsulation coats for entrapment of garden cress phenolic-rich extract: improved thermal stability, storage stability, antioxidant and antibacterial activities

**DOI:** 10.1007/s10068-022-01171-3

**Published:** 2022-09-20

**Authors:** Azza M. Abdel-Aty, Amal Z. Barakat, Saleh A. Mohamed

**Affiliations:** Molecular Biology Department, National Research Centre, Dokki, Cairo, Egypt

**Keywords:** *Lepidium sativum*, Garden cress 6-day sprouts, Encapsulation efficiency, Phenolic content, Storage stability

## Abstract

The obtained garden cress 6-day sprouts phenolic-rich extract (GCSP) contained efficient health-promoting antioxidant-phenolic compounds. To improve the stability, bioavailability, and functional properties of these valuable phenolic compounds, GCSP was encapsulated by freeze-drying technique using different ratios of garden cress gum (GG) and maltodextrin (M) in the absence and presence of sonication (S). The prepared S/GG-microcapsule retained the highest phenolic content (95%), antioxidant activity (141.6%), and encapsulation efficiency (98.2%). It displayed the highest bio-accessibility of GCSP-phenolic compounds in simulated intestine fluid (87%) and demonstrated the greatest storage-stability at 40 °C for 60 days. S/GG-microcapsule possessed better physical properties including moisture, solubility, swelling, and morphological structures using SEM. The main spectral features, crosslinking, and improved thermal stability were demonstrated for S/GG-microcapsule using FTIR and thermogravimetric analyses. S/GG-microcapsule demonstrated much greater antibacterial activity than GCSP against pathogenic bacteria. S/GG-microcapsule can be added to different food products to improve their antioxidant and antibacterial properties.

## Introduction

Consumers' awareness of the vital relationship between food and health is increasing the demand for functional food products. Natural phenolic compounds are valuable compounds, extracted mainly from plants, and have free radical scavenging activity against the harmful radical oxygen species as well as complexing properties towards proteins (Munin and Edwards-Lévy, 2011; Mohamed et al., 2016). Thus, the addition of these phenolic compounds to the human diet could remarkably prevent several dangerous diseases such as inflammation and diabetes (Abdel-Aty et al., 2019c; Barakat et al., 2020a, 2020b). Besides, they have anti-aging properties in cosmetic products, therapeutic effects against viper venoms, and antimicrobial activity (Agrawal and Kaur, 2010; Abdel-Aty et al., 2018; Saleh et al., 2018). Unfortunately, the antioxidant-phenolic compounds are easily oxidizing by some environmental factors like light, oxygen, and moisture due to the unsaturated bonds in their structures leading to considerable loss of their potent biological properties. Moreover, many of these compounds have unwanted odors and/or unpleasant tastes, reducing their use in food or oral medications (Fang and Bhandari 2010). In addition, these compounds suffered from instability during food processing, shelf storage, or in the gastrointestinal tract resulting in a reduction of their antioxidant activity and health benefits (Nedovic et al., 2011; Al-Najada and Mohamed, 2014; Ray et al., 2016; Alali et al., 2018). Therefore, the antioxidant-phenolic compounds require a special formulation to maintain their structural integrity, mask the taste, and increase water solubility and bioavailability for providing their benefits to consumers (Khalid et al., 2017).

Encapsulation is an attractive technique that protects antioxidant-phenolic compounds by covering them in a coating material as a resistant layer. This technique increases the shelf-life, stability in the gastrointestinal tract or food processing, and bioavailability of the phenolic compounds, besides masking their unwanted odor, taste, and color. In addition, some previous studies reported that the encapsulation technology increased the intestinal uptake of phenolic compounds, and improved their bioactivity (Song et al., 2014).

The freeze-drying process is one of the most widely used encapsulation techniques for phenolics. This drying technique retains most of the initial functional properties of the antioxidant-phenolic compounds (Ceballos et al., 2012). The wall materials also affect the encapsulation efficiency of phenolics. Maltodextrin is an example of common coating material used in the encapsulation of active compounds. It is very effective for the encapsulation of bioactive compounds (Fernandes et al., 2014). Garden cress gum, extracted from garden cress seeds, is a hetero-polysaccharide that possessed good emulsifying properties and is used for fish oil and orange peel oil encapsulation (Razmkhah et al., 2016; Kavousi et al., 2017; Dehghan et al., 2020). Also, it is considered a natural nutraceutical with protective action against enter-colitis (Akl et al., 2021).

*Lepidium sativum* L. (garden cress) is a fast-growing plant native to Egypt and currently, it is sown in many countries as a significant medicinal plant. Garden cress seeds are a rich source of fats, proteins, dietary fibers, minerals, and vitamins. Further, these seeds possess various medicinal effects including hypoglycemic, gastro-protective, diuretic, expectorant, bronchitis, bone fractures, dyspepsia, and laxative (Diwakar et al., 2010). Moreover, they possessed phenolic compounds with the highest antioxidant activity, which stopped the degenerative damage caused by oxidative stress and have antimicrobial properties (Shirwaikar et al., 2011; Abdel-Aty et al., 2019a). Recently, we upgraded the total phenolic and flavonoid contents and antioxidant activity of the garden cress seeds several folds besides producing new phenolic compounds during the germination process. The garden cress 6-day sprouts phenolic-rich extract (GCSP) could be used as a dietary supplement as well as in different applications (Abdel-Aty et al., 2019b). However, the encapsulation of GCSP for conservation of their therapeutic and antioxidant properties is not reported before. Thence, the present study aims to evaluate the efficiency of two coating materials maltodextrin and gum of garden cress (unmixed and mixed at different ratios, and with and without sonication) for retention of GCSP and preserve their biological properties by using the freeze-drying encapsulation technique.

## Materials and methods

### Phenolic-rich extract of 6-day garden cress sprouts (GCSP) preparation

A large amount of GCSP was prepared as previously described by Abdel-Aty et al. (2019b). Briefly, the garden cress 6-day sprouts were oven-dried at 50 °C and then extracted in 80% methanol under overnight shaking at 150 rpm and filtered through filter paper (Whatman 1). The filtrate (GCSP) was concentrated under vacuum in a rotary evaporator at 45 °C, freeze-dried at – 55 °C, and then stored at – 20 °C for further use in the encapsulation experiments.

### Garden cress gum preparation

Garden cress whole seeds were mixed and soaked in distilled water at a ratio of 1:30 (seed: water w/v) for 1 h at 40 ºC. Then the obtained mucilage was separated by filtration according to Karazhiyan et al. (2011). Three volumes of ethanol were added to one volume of mucilage and the precipitate was collected, dried, and ground (Razavi et al., 2009).

### Encapsulation process

The GCSP was encapsulated using maltodextrin DE 8–15 (M) and the prepared garden cress gum (GG) as coating materials. M and GG, each weighing 100 mg, were individually dissolved in 100 ml distilled water at a ratio of 1:1 (w/v) at room temperature with stirring at 700 rpm. Each coating material solution was prepared one hour before the encapsulation process. The freeze-dried GCSP was mixed with the coating materials at four different cores in the following ratios: M: GCSP (10:1), M + GG: GCSP (8 + 2:1), GG + M: GCSP (8 + 2:1), and GG: GCSP (10:1). The mixtures were stirred at 1500 rpm for 1 h to obtain a homogeneous dispersion of wall material with GCSP. The half volume of each homogenous suspension was sonicated (S) for 10 min and the second half was left without sonication. All the prepared samples were frozen at− 80 °C and then freeze-dried at -55 ºC. The obtained microcapsules were kept at 4 °C.

### Total phenolic content determination

The phenolic content of GCSP before and after the encapsulation process was measured by the Folin-Ciocalteu reagent according to Velioglu et al. (1998). Firstly, ten mg of either GCSP or the prepared microcapsules were dispersed/extracted in 1 ml of ethanol, acetic acid, and water (50:8:42) mixture for 1 min. The mixture was vortexed for 1 min and filtered by a 0.5 µm filter (Saenz et al., 2009). The obtained extract (10–50 µl), Folin-Ciocalteu reagent (100 µl), and distilled water (890–850 µl) were mixed and incubated for 5 min. A 500 µl of 20% sodium carbonate was added and incubated for 30 min. Absorbance was measured at 750 nm. The phenolic content was calculated as mg gallic acid equivalent (GAE)/g DW.

### Total antioxidant activity using DPPH free radicals-assay

1,1-Diphenyl-2-picrylhydrazyl (DPPH) was used for the detection of the antioxidant activity of GCSP before and after the encapsulation process according to Ao et al. (2008). Firstly, according to Saenz et al (2009), the phenolic content of either GCSP or the prepared microcapsules was extracted as mentioned above. Then, 0.9 ml of 0.1 mM DPPH dissolved in methanol and 0.1 ml of the obtained extract were incubated for 30 min in the dark at room temperature. The absorbance was measured at 517 nm. The antioxidant activity was calculated according to Eq. (1):

1 $${\text{DPPH}}\;{\text{scavenging}}\;\% \; = \;\left[ {\left( {{\text{O.D. control}}} - {\text{O.D. sample}} \right)/{\text{O.D. control}}} \right]\; \times \;100.$$

Total antioxidant activity based on the Trolox equivalent (TE) standard curve was calculated.

#### Surface phenolic content and encapsulation efficiency determination

For determination of the surface phenolic content (SPC), 10 mg of each prepared microcapsule was extracted in a mixture of 1 ml of ethanol and methanol (1:1) and filtered (Cilek et al., 2012). The obtained SPC phenolic content was measured as described above by the Folin-Ciocalteu method. The encapsulation efficiency (EE) of the prepared microcapsules was measured according to Eq. (2); (Cilek et al., 2012).

2 $${\text{EE }}\% \; = \;{\text{EPC}}/{\text{TPC}}\; \times \;{1}00\; = \;\left( {{\text{TPC}} - {\text{SPC}}/{\text{TPC}}} \right)\; \times \;{1}00$$

where EPC is encapsulated phenolic content (mg GAE), TPC is total phenolic content (mg GAE), SPC is surface phenolic content of the prepared microcapsule (mg GAE).

### In vitro simulated gastrointestinal digestion

The amount of the phenolic content released from the prepared microencapsulates was determined under simulated gastric fluid (SGF) and simulated intestinal fluid (SIF) conditions (Noor et al., 2022). For SGF, each prepared microcapsule was dispersed in 10 ml distilled water containing 0.3 mM NaCl, 4 mg pepsin, and 0.1 M HCL at pH 2.0 and incubated at 37 ºC for 2 h with shaking at 100 rpm. After gastric digestion, the pH of the solution was changed to 7.3, pancreatin was added and the solution was incubated for 2 h at 37 ºC. At the end of each digestion step, the sample was centrifuged for 20 min at 5000 rpm, neutralized, the enzyme activity was inhibited and the phenolic content was determined using the Folin-Ciocalteu method. The bio-accessibility index was measured using Eq. (3):

3 $${\text{Bio-accessibility}}\;{\text{index}}\; = \;\left( {{\text{P}}_{{{\text{release}}}} /{\text{P}}_{{{\text{total}}}} } \right)\; \times \;100$$

where P_release_ is the phenolic content released from the microcapsule (mg GAE), P_total_ is the total phenolic content found in this microcapsule (mg GAE).

### Thermal storage stability

According to the method of Vu et al. (2020), the stability of the phenolic compounds of the GCSP before and after the encapsulation process was evaluated during 60 days of incubation at 40 ºC. The powders of GCSP and the prepared microcapsules were packed in brown plastic vials (20 mg/vial) and were incubated at 40ºC. Samples were taken every 15 days, and the total phenolic content was measured as mentioned above.

## Physical characterizations

### Determination of moisture, solubility, and swelling

According to Vu et al. (2020), the moisture content of microcapsules was examined by drying microcapsules at 105 °C overnight and then measured by Eq. (4):

4 $${\text{Moisture}}\;{\text{content}}\;\% \; = \;\left( {{\text{W}}_{{\text{b}}} \; - \;{\text{W}}_{{\text{a}}} } \right)/{\text{W}}_{{\text{b}}} \; \times \;100$$

W_b_ and W_a_ are the weights in mg of each capsule before and after drying, respectively.

The solubility of the prepared microcapsules was examined as described by Vu et al. (2020). One hundred mg of each microencapsulate was mixed in 15 ml of deionized water, vortexed for 1 min, incubated at 37 °C for 30 min, and centrifuged at 5,000 rpm for 5 min. Each supernatant was dried at 105 °C overnight. The solubility % was calculated by Eq. (5):

5 $${\text{The}}\;{\text{solubility}}\;\% \; = \;\left( {{\text{w}}_{1} \; - \;{\text{w}}_{2} } \right)/{\text{w}}_{1} \; \times \;100.$$

where the w_1_ is the initial weight in mg of capsulate and the w_2_ is the supernatant weight in mg after drying.

The swelling of the prepared microcapsules was examined according to Taheri et al. (2020). Fifty mg of each prepared microencapsulate was immersed in distilled water for 3 h at room temperature. The samples were removed from water by vacuum filtration and re-weighted after removal of the out-excess water using a filter paper. The swelling % was calculated by Eq. (6):

6 $${\text{The}}\;{\text{swelling}}\;\% \; = \;\left( {{\text{w}}_{2} \; - \;{\text{w}}_{1} } \right)/{\text{w}}_{1} \; \times \;100$$

where the w_1_ is the initial weight in mg of the capsulate and the w_2_ is the weight in mg of the swollen microencapsulate after 3 h.

### Surface morphology

The surface morphology study of the pure coating materials (M and GG-gum) and the prepared GCSP microcapsules was analyzed using Holland Field Emission Scanning Electron Microscope (FE-SEM, QUANTA FEG250) with an accelerating voltage of 20 kV.

### Fourier Transform Infrared analysis (FTIR-analysis)

The FTIR Spectrometer (Bruker ALPHA-FTIR-Spectrometer) was employed to take spectra of the prepared S/GG-microcapsule and its coating material (GG-gum). Platinum-attenuated reflection was used at a wave range of 400–4000 cm^1^.

### Thermal analysis

The thermal properties of the prepared S/GG-microcapsule and its coating material GG-gum were evaluated by non-isothermal thermogravimetric (TGA) and differential thermogravimetric (DTG) analyses. Sample runs were performed at a constant heating rate of 10 °C/min from 40 °C to 800 °C.

### Antibacterial activity evaluation

Broth dilution and colony counting methods (Shetta et al., 2019) were implicated to evaluate the antibacterial potency of GCSP and the prepared S/GG- microencapsulate against *Escherichia coli* O157-H7 ATCC 51,659, *Salmonella typhi* ATCC 15,566 and *Staphylococcus aureus* ATCC 13,565, *Bacillus subtilis* BTN7A as gram-negative and gram-positive human enteric pathogenic bacterial strains, respectively. The bacterial strains were obtained from the Food toxins and Contaminants Department, National Research Centre, Giza, Egypt. The pathogenic bacteria were grown in Muller-Hinton broth (MHB) at 37 °C with 180 rpm shaking. After overnight incubation, 100 µl of each pathogenic strain was cultured again in falcon tubes containing one ml MHB mixed with different concentrations of either GCSP or S/GG-microencapsulate, and the incubation was conducted overnight at 37 °C under shaking. Each falcon tube suspension was spread on Mueller–Hinton agar plates, cultivated at 37 °C overnight and counted the number of bacterial colonies. The antibacterial activity was evaluated using the minimum bactericidal concentration (MBC), which is defined as the minimum concentration killing 99.9% or more of the initial inoculation.

### Statistical analysis

The data were statistically analyzed by a one-way ANOVA. The data were considered as means ± S.D. (n = 6).

## Results and discussion

### Total phenolic content, total antioxidant activity, and encapsulation efficiency

The selection of coating material has a considerable impact on the encapsulation efficiency as well as the solubility, stability, and quality of the final product. In this study, maltodextrin (M) and garden cress gum (GG-gum) as coating materials and their combination at different ratios and sonication process (S) were used for encapsulation of the phenolic extract of the garden cress 6-day sprouts (GCSP) using the freeze-drying technique. Table 1 screens the total phenolic content and the antioxidant activity of the GCSP before and after the encapsulation process as well as the encapsulation efficiency (EE). The values of the total phenolic contents for the prepared microencapsulates (7.6–28.5 mg GAE/g DW) were significantly lower (*P* < 0.01) than the phenolic content of the unprocessed extract (30.0 mg GAE/g DW). The reduction in the phenolic content during the encapsulation process may occur due to many factors such as the drying process used. During the freeze-drying process, the phenolic active compounds interfere with the structure of one or more polymers resulting in the loss of some phenolic compounds. Besides, the grinding of freeze-dried products may lead to the degradation of some phenolic compounds and exposure to some oxidation reactions. Similar results were obtained in many studies that used the freeze-drying technique for encapsulation of phenolic compounds (Franceschinis et al., 2014; Ballesteros et al., 2017; Akdeniz et al., 2018).

**Table 1:** Total phenolic content (TPC), surface phenolic content (SPC), encapsulation efficiency % (EE) and total antioxidant activity (TAA) of garden cress 6-day sprout phenolic extract encapsulated with different wall materials added in different ratios using the freeze-drying technique

Sample	Wall: GCSP ratio	TPC mg GAE/g DW	SPC mg GAE/g DW	EE (%)	TAA mg TE/g DW Using DPPH	TAA retention %
GCSP	–	30.0 ± 1.4^a^	–	–	47.3 ± 2.0^a^	100.0^a^
M	10:1	7.6 ± 0.3^b^	1.5 ± 0.05^a^	80.3^a^	13.5 ± 0.7^b^	28.5^b^
S/M	10:1	9.8 ± 0.4^c^	1.4 ± 0.04^b^	85.7^b^	19.0 ± 1.0^c^	40.2^c^
M + GG	8 + 2:1	12.0 ± 0.6^d^	1.3 ± 0.07^c^	89.2^c^	22.4 ± 1.0^d^	47.4^d^
S/M + GG	8 + 2:1	14.5 ± 0.7^e^	1.1 ± 0.06^d^	92.4^d^	30.0 ± 1.3^e^	63.4^e^
GG + M	8 + 2:1	20.5 ± 1.0^f^	1.0 ± 0.05^e^	95.1^d^	42.5 ± 2.0^f^	90.0^f^
S/GG + M	8 + 2:1	23.0 ± 1.1^ g^	0.8 ± 0.03^f^	96.5^d^	46.0 ± 2.2^a^	97.2^a^
GG	10:1	25.6 ± 1.1^ h^	0.7 ± 0.02^ g^	97.3^e^	61.5 ± 2.7^ g^	130.0^ g^
S/GG	10:1	28.5 ± 1.2^ k^	0.5 ± 0.01^ h^	98.2^e^	67.0 ± 2.5^ k^	141.6^ k^

Values are presented as means ± SD (n = 6); results in the same column with different superscripts are significantly different at (*p* < 0.01)

*GAE* gallic acid equivalent, *TE* trolox equivalent, *GCSP* garden cress 6-day sprouts phenolic extract, *M* maltodextrin, *GG* garden cress gum, *S* sonicated sample

Among all the prepared microencapsulates, M-microencapsulate with a ratio of 10 M: 1 GCSP showed the lowest phenolic content and EE percentage (7.6 mg GAE/g DW and 80.3%, respectively). However, the retention of phenolic content gradually increased incompatible with increasing the GG-gum concentration in the prepared microencapsulates. Where, moderate retention phenolic contents and EE percentages (12.0–23.0 mg GAE/g DW and 89.2–96.5%, respectively) were observed for microencapsulates containing a mixture of M and GG-gum (M/GG, SM/GG, GG/M and SGG/M microencapsulates). Finally, the highest phenolic content and EE percentage (28.5 mg GAE/g DW and 98.2%, respectively) were observed for S/GG-microencapsulate with a ratio of 10 GG-gum: 1 GCSP. Under the same conditions, significant changes (*P* < 0.01) were observed between the phenolic contents retained in the sonicated and un-sonicated microencapsulate. These results confirmed that wall material is a key factor for encapsulating phenolic compounds. In comparison with various previous reports, lower encapsulation efficiency of 27–75% was reported for M-microencapsulates of the blueberry juice phenolic extract, coffee grounds phenolic extract, cactus pear juice extract, black carrot extract, black carrot juice, and pomegranate juice (Ersus and Yurdagel, 2007; Saenz et al., 2009; Murali et al., 2015; Wilkowska et al., 2016; Ballesteros et al., 2017). Similar to our findings, the M and gum Arabic microencapsulates of the banana peel phenolic compounds gave 93 and 92% efficiency, respectively (Vu et al., 2020), as well as star fruit pomace M-microencapsulates showed encapsulation efficiency of 78–97% (Saikia et al., 2015).

For antioxidant activity used DPPH assay, the M and S/M-microencapsulates showed a significant decrease (*P* < 0.01) in the total antioxidant activity (13.5 and 19 mg TE/g DW) with retention percentages of 28.5 and 40.2%, respectively compared to the initial antioxidant capacity of the GCSP (47.3 mg TE/g DW). Moderate values of total antioxidant activity and retention percentage (22.4–46.0 mg TE/g DW and 47.4–97%, respectively) were obtained for microencapsulates containing a mixture of M and GG-gum as wall materials. While the prepared microencapsulates GG and S/GG recorded significantly higher (*P* < 0.01) total antioxidant activity values of 61.5 and 67.0 mg TE/g DW with retention percentages of 130 and 141.6%, respectively compared to the initial extract as shown in Table1. Such observation may be due to the concentration of some antioxidant-phenolic compounds increasing after the encapsulation process, which have a higher susceptibility to the DPPH free radicals. Garofulic et al. (2017) found that the concentration of the quercetin-3-glucoside was significantly higher in encapsulated sour cherry with gum Arabic than in unprocessed extract. In addition, the H-microencapsulate of blackberry phenolic compounds showed a higher concentration of catechin compared to the original extract (da Rosa et al., 2014). The encapsulated flours showed higher total phenolic content and antioxidant capacity than unprocessed flour (Ahmed et al., 2010). Many reports showed that some extracted gums from plants have a potent antioxidant activity such as peach gum, *Albizia stipulate* gum, and Arabic gum (Yao et al., 2013; Thanzami et al., 2015; Hu et al., 2018). In addition, the GG-gum possessed a potent antioxidant activity (Akl et al., 2021). It was also observed that all sonicated microencapsulates showed a significantly higher (*P* < 0.01) retention of total antioxidant activity compared to the un-sonicated microencapsulates. It can be observed that employing GG-gum as a coating material for encapsulating the GCSP retained the greatest amount of phenolics and antioxidants. However, the lowest phenolic content and total antioxidant activity were obtained when the M was used alone to encapsulate the GCSP.

The M is a D-glucose polymer, while GG-gum is a heteropolysaccharide with a little amount of protein in addition to the sugar content. The protein content in the GG-gum structure possessed a hydrophobic character which is responsible for the good emulsifying properties of the GG-gum, besides the hydrophilic properties of its sugar content (Razmkhah et al., 2016). Both hydrophobic and hydrophilic properties of the GG-gum may describe the higher retention of the antioxidant-phenolic compounds in GG-microencapsulates compared to the M- microencapsulates.

### In vitro simulated gastrointestinal digestion

**Fig. 1a:**
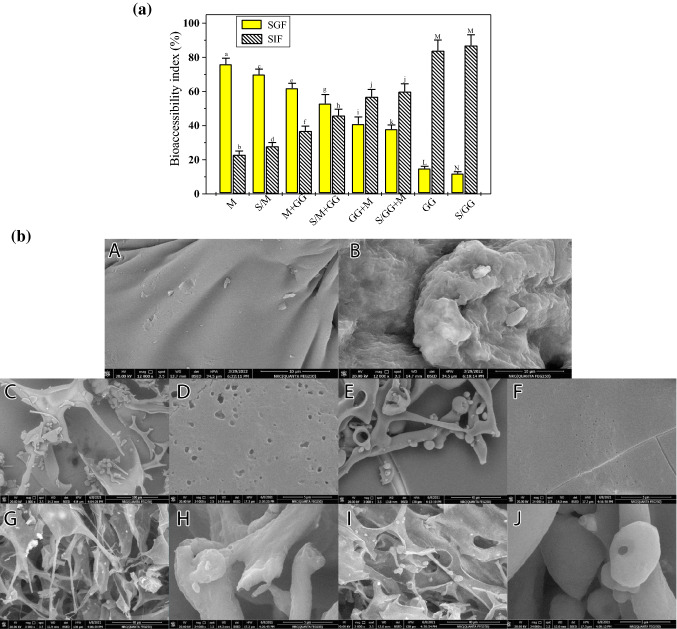
Release rate/bio-accessibility index percentages of the GCSP-phenolic compounds from the prepared microencapsulates under simulated gastric fluid (SGF) and simulated intestine fluid (SIF). M: Maltodextrin-microencapsulate; GG: Garden cress gum-microencapsulate; S: Sonicated sample; GCSP: Garden cress 6-day sprouts phenolic extract. Values are presented as means ± SD (n = 6); Different letters are statistically different at (*P* < 0.01). Figure 1b. SEM micrographs of pure coating materials (maltodextrin and garden cress gum) (A and B), M-microcapsule (C and D), S/M- microcapsule (E and F), GG-encapsulate (G and H), and S/GG-encapsulate (I and J). SEM graphs were taken at low and high magnifications. M: Maltodextrin- microencapsulate; GG: Garden cress gum-microencapsulate; S: Sonicated sample

Figure 1a lists the bio-accessibility index percentages of the GCSP-phenolic compounds of the prepared microencapsulates after simulated in vitro gastrointestinal digestion. Simulated gastric fluid (SGF) was evaluated at 37° C and pH 2.0, whilst simulated intestinal fluid (SIF) was evaluated at pH 7.3. In SGF, the M and S/M-microencapsulates showed a sudden release of their phenolic compounds after gastric digestion with a bio-accessibility index of 76 and 70%, respectively. Additionally, with increasing GG-gum concentration, the release of phenolic compounds from the prepared microencapsulates dropped gradually, with a bio-accessibility index ranging from 62 to 38%. The GG and S/GG-microencapsulates revealed the least phenolic compounds-release of 15 and 12%, respectively, of the initial phenolic contents after gastric digestion. However, following intestinal digestion, the phenolic compounds released by the GG and S/GG-microencapsulates were the highest, with bio-accessibility index percentages of 84 and 87%, respectively, and the M and S/M-microencapsulate released the lowest phenolic compounds with an index of 23 and 25%, respectively (Fig. 1a). This finding suggests that the phenolic compounds in both GG- and S/GG-microcapsules are highly bio-accessible in the intestinal phase, implying that the GG-gum protected the GCSP-phenolic compounds from degradation under gastric conditions and hence improved their availability in the intestinal conditions. The GG-gum may be poorly digested by pepsin in gastric fluid (at pH 2.0), but it can be easily digested by pancreatin in intestinal fluid (at pH 7.3), making the GG-gum a suitable coat for controlled delivery of the GCSP-phenolic compounds. Thus, the GG- and S/GG-microcapsules can be incorporated into various food products to protect their active phenolic content from degradation and preserve their biological activities. Similar controlled-release profiles were previously reported (Yi et al., 2015; Tumbas Saponjac et al., 2016; Vulic et al., 2019; Noor et al., 2022).

### Storage stability of the phenolic content

Changes in the total phenolic contents of both unprocessed GCSP and encapsulated powders stored at 40 °C for 2 months are listed in Table 2. The phenolic contents of the GCSP and the prepared M and S/M-microencapsulates significantly decreased (*P* < 0.01) from (30.5, 7.9, and 10.0 mg GAE/g DW, respectively) to reach (8.0, 4.1, and 6.5 mg GAE/g DW, respectively), with a loss percentage about 74, 48 and 35%, respectively after 60 days of incubation at 40 °C. This result appeared that phenolic content in the M-encapsulates is not protected, and is readily available to interact with the environmental moisture and oxygen. The decrease of phenolics from 10.0% to 17.5% occurred for M-Melissa, M-Tussilago, and M-Fadogia capsules after 6 months of incubation at 40 °C (Sansone et al., 2011). However, retention of the phenolic content remained constant in the prepared microencapsulates that contain a mixture of the GG-gum and M as coating materials. The phenolic content of the GG- and S/GG- microencapsulates significantly increased (*P* < 0.01) from 25.0 and 28.2 mg GAE/g DW to reach 30.8 and 35.0 mg GAE/g DW after 60 days of incubation at 40 °C, respectively. This increase in phenolic content in GG and S/GG-microencapsulates may be due to the effect of the elevated temperature on the hydrolysis of phenolic-glycosides and the production of more free phenolic compounds which have a high sensitivity toward Folin-Ciocalteu reagent (Turkmen et al., 2005). Robert et al. (2015) showed that the retention of the phenolic content of the purple cactus pear remained constant for 28 days of incubation at 60 °C and then increased in the three microencapsulates (~ twofold) after 50 days of incubation at 60 °C. Vu et al. (2020) reported no change in phenolic content of all M and gum Arabic encapsulates that were prepared for banana peel phenolic extract after 30 days of incubation at 40 °C. The M and gum Arabic encapsulates of the cherry juice phenolic extract retained 90% of the phenolic content after 33 days of incubation at 38 °C (Sanchez et al., 2015). From these findings, as expected, the storage stability was strongly dependent on the coating material properties such as embedding capacity and core retention ability as well as encapsulation efficiency. Therefore, it can be concluded that the GG-gum is a good protector for the phenolic compounds of the GCSP and the prepared GG and S/GG-microencapsulates are suitable for long-term storage even under hard storage conditions.

**Table 2:** Storage stability assessment of garden cress 6-day sprouts phenolic extract before and after encapsulation process during 60 days at 40 °C

Sample	TPC (mg GAE/g DW)				
	Time (days)				
	0	15	30	45	60
GCSP	30.5 ± 1.3^a^	20.0 ± 1.2^b^	14.5 ± 0.85^c^	10.2 ± 0.5^d^	8.0 ± 0.30^e^
M	7.6 ± 0.32^a^	5.1 ± 0.25^b^	4.7 ± 0.21^c^	4.0 ± 0.11^d^	4.1 ± 0.14^d^
S/M	10.0 ± 0.31^a^	8.8 ± 0.23^b^	7.2 ± 0.25^c^	6.8 ± 0.12^d^	6.5 ± 0.21^d^
M + GG	12.2 ± 0.53^a^	12.3 ± 0.46^a^	12.5 ± 0.75^a^	12.2 ± 0.63^a^	12.1 ± 0.52^a^
S/M + GG	14.2 ± 0.71^a^	14.4 ± 0.53^a^	14.5 ± 0.41^a^	14.5 ± 0.50^a^	14.2 ± 0.11^a^
GG + M	20.3 ± 1.0^a^	21.0 ± 1.1^a^	21.0 ± 1.2^a^	20.5 ± 1.2^a^	20.4 ± 0.82^a^
S/GG + M	23.5 ± 1.1^a^	23.6 ± 1.3^a^	23.5 ± 1.1^a^	22.9 ± 1.2^a^	22.8 ± 0.71^a^
GG	25.4 ± 1.2^a^	25.5 ± 0.87^a^	25.5 ± 1.2^a^	27.0 ± 1.1^b^	30.8 ± 0.75^c^
S/GG	28.4 ± 1.3^a^	28.5 ± 1.2^a^	28.6 ± 1.20^a^	32.1 ± 1.1^b^	35.0 ± 0.9^c^

Values are presented as means ± SD (n = 6)

*GCSP* Garden cress 6-day sprouts phenolic extract

*TPC* total phenolic content, *GAE* gallic acid equivalent, *M* maltodextrin, *GG* garden cress gum, *S* sonicated sample. Different letters in the same raw are statistically different at (*P* < 0.01)

### Physical properties

#### Moisture, solubility, and swelling properties

Table 3 shows the moisture, solubility, and swelling percentage of the GG and S/GG-microencapsulates compared to M and S/M-microencapsulates. The moisture content of the GG and S/GG-microencapsulates (6.3 and 4.6%, respectively) was significantly lower than the moisture content of the M and S/M microencapsulates (11.9 and 8.5%, respectively). The variation in encapsulate moisture content is determined by the coating materials used (Sahin-Nadeem et al., 2013). In addition, higher moisture content causes particle stickiness and an increase in flow resistance (Arepally and Goswami 2019).

**Table 3:** Moisture, solubility and swelling % of the prepared M, S/M, GG, and S/GG microencapsulates

Encapsulate	Moisture (%)	Solubility (%)	Swelling (%)
M	11.9 ± 0.32^a^	95 ± 1.0^a^	102 ± 2.11^a^
S/M	8.5 ± 0.21^b^	99 ± 1.1^a^	105 ± 3.22^b^
GG	6.3 ± 0.40^c^	47 ± 4.2^b^	141 ± 4.8^c^
S/GG	4.6 ± 0.15^e^	79 ± 4.2^c^	155 ± 4.2^b^

Values are presented as means ± SD (n = 6). Different letters in the same column are statistically different at (*P* < 0.01)

*M* Maltodextrin-encapsulate, *GG* Garden cress gum-encapsulate, *S* sonicated sample

The solubility of the M and S/M-microencapsulates (95 and 99%, respectively) was greater than the GG and S/GG- microencapsulates (47 and 79%, respectively). The Sonication process worked to increase the solubility of both M- and GG-microencapsulates. This result explains the sudden release of the phenolic content from the M-microencapsulates in gastric solution compared to the uniform/controlled release of phenolics from GG-microencapsulates as observed above. The hydrophilic character of M is responsible for its higher solubility (Arepally and Goswami 2019).

The swelling properties of the GG- and S/GG-microencapsulates (141and 155%, respectively) were better compared to the M and S/M-microencapsulates (102 and 105%, respectively). This result is due to the gelling nature of the GG-gum that swells when it absorbs water and forms gel. The gelling ability/gel formation property of GG-gum is a phenomenon involving the formation of 3-dimensional network chains that traps water and form a gel. In addition, the gelling nature of the GG-gum leads to the conservation of the structure and properties of food (Naji et al., 2012). This explains the high ability of GG and S/GG-microcapsules to preserve the phenolic compounds of GCS compared to M and S/M-encapsulates.

#### SEM-Morphology

SEM images for coating materials, M and GG-gum, and the prepared M, S/M, GG, and S/GG-microencapsulates are shown in Fig. 1b. Both coating materials showed marked differences in surface morphology. M image revealed a smooth surface morphology while the GG-gum showed an irregular and rough surface morphology (Fig. 1b, A and B). Freeze-drying changed the morphology of both coating materials, resulting in irregular rough surface structures (Mahdavee Khazaei et al., 2014). M-microencapsulate revealed irregular particle morphology with a rough surface and micropores were observed within the particle walls, which appeared to be hollow (Fig. 1b, C and D). These micropores absorbed the phenolics and they allow the phenolic compounds to retain in microencapsulates. Visible open micropores on the particle surface can easily lose the phenolic compounds as a core material from the coating material. This may explain why the M-microencapsulate recorded the lowest phenolic content retention and storage stability. While the prepared S/M-microencapsulate showed irregular particle shapes with a smooth surface and few micropores (Fig. 1b, E and F), suggesting that the sonication process improved the structure and efficiency of the prepared microencapsulates. Moreover, the results revealed that both GG and S/GG-microencapsulates appeared sawdust-like structures without micropores (Fig. 1b, G, H, and I, J, respectively). However, the particles of the S/GG- microencapsulate showed smoother surfaces. Such structures improve the surface area of the coating material, allowing more antioxidant-phenolic compounds to be retained and producing microencapsulates with a higher storage capacity. Therefore, the prepared S/GG-microcapsule is more appropriate for preserving the antioxidant-phenolic compounds of GCSP.

### FTIR-spectra analysis

**Fig. 2:**
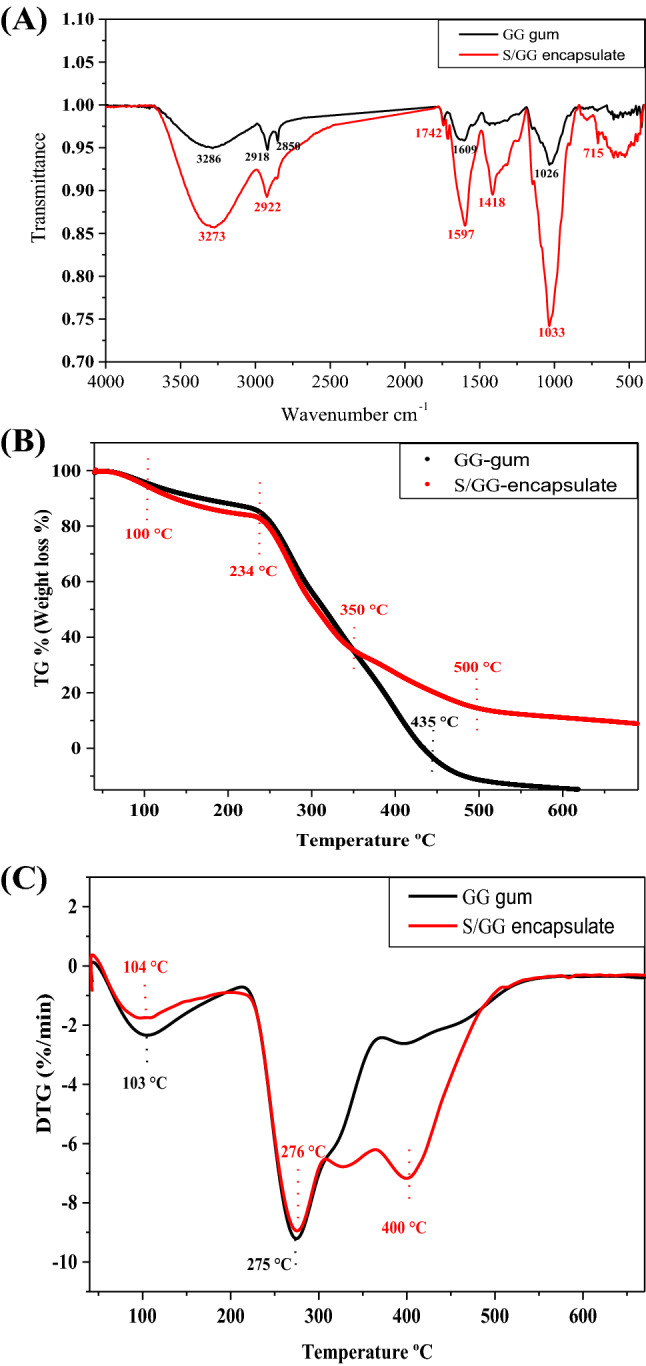
(**A**) FTIR spectra of the prepared S/GG-microencapsulate and its coating material garden cress gum (GG-gum). TGA curves (**B**) and DTG curves (**C**) of the prepared S/GG-microencapsulate and its coating material garden cress gum (GG-gum)

As can be seen in Fig. 2A, the FTIR spectra provided the main GG-gum characteristic peaks matched to O–H stretching band at 3286 cm^−1^ (for alcohol and carboxylic acid groups), and C–H_2_ symmetric and asymmetric stretching bands at 2918 and 2850 cm^−1^, derived from carbohydrates and sugars, respectively. In addition, C=O stretching band at 1609 cm^−1^ (for carboxylic acid groups) and negative carbonyl (C–O) stretching bands at 1026 cm^−1^. These peaks were identified in the garden cress gum as a polysaccharide natural product (Kavousi et al., 2017). Following the encapsulation process, the prepared S/GG-microencapsulate spectra showed vibrations of several O–H groups at 3273 cm^−1^(broadband with high intensity), aromatic C–C stretching at 1418 cm^−1^, and rocking of CH_2_ at 715 cm^−1^, all related to phenolic compound structures (Lucarini et al., 2020). A shoulder band C=O at 1742 cm^−1^ was observed due to conjugation of ester-groups which occurs during the crosslinking reactions (Paulino et al., 2010). Moreover, O–H, C–H_2,_ C=O, and C–O peaks were shifted from 3286, 2918, 1609, and 1026 to 3273, 2922, 1597, and 1033 cm^−1^, respectively, with high intensity, after encapsulation and sonication processes. Overall, the molecular structure of GG-gum was altered due to the encapsulation and sonication processes, as well as the cross-linking between GG gum and GCSP occurred.

### Thermal stability

The thermal stability of the S/GG-microencapsulate, as the best-obtained capsule properties, was evaluated using DTG and TGA analyses in comparison to its coating GG-gum. TGA curves of GG-gum and S/GG-microencapsulate were divided into two distinct processes including the dehydration and decomposition processes (Fig. 2B). For GG-gum, the dehydration process started around 100 to 232 °C, while for S/GG encapsulation started from 100 to 234 °C with a low weight/mass loss of 15 and 16%, respectively. Above 100 °C, a small amount of water/moisture content entrapped in the molecules evaporated and is responsible for this initial weight/mass loss. The S/GG-microencapsulate initial decomposition stage was observed around 250 to 350 and continues till 500 °C. Whereas, the initial decomposition stage of the GG-gum was observed around 250 to 435 °C. The S/GG-microencapsulate showed a lower weight/mass loss than its coating material (GG-gum), confirming a successful encapsulation process has occurred. Additionally, the prolonging in the initial decomposition stage suggests that the polymer structure was widened. Thermal transition showed that S/GG- microencapsulate is more thermally stable which retained 22 and 11% of its weight at 435 and 650 °C, respectively, while the GG-gum fully degraded at 435 °C (Fig. 2B). This transition in the locations is attributed to the depolymerization of the interfered materials. Following encapsulation, mainly within the gum, the samples' thermal stability improved (Ballesteros et al., 2017). The thermal decomposition of spent coffee ground-phenolic compounds encapsulated with maltodextrin (> 190 °C) was lower than that reported for encapsulated with gum Arabic (> 225 °C) (Ballesteros et al., 2017). In DTG curves, the maximum decomposition rate for GG-gum was detected at one temperature of 275 °C while S/GG-microencapsulate showed two-step decomposition temperatures of 276 and 400 °C (Fig. 2C). From these findings, the increase in initial and final decomposition temperatures of the S/GG-microencapsulate over the GG-gum coating material confirms the improvement in the thermal stability of the prepared S/GG-microencapsulate.

### Antibacterial activity evaluation

The antibacterial efficacy of S/GG- microencapsulate in comparison with GCSP was examined against some enteric pathogenic bacteria. The S/GG- microencapsulate demonstrated considerably higher (*p* < 0.01) antibacterial activity against all the examined bacteria, with MBC values ranging (0.7–1.3 mg/ml), as compared to GCSP (2.7–3.5 mg/ml) and Amoxicillin (1.2–2.5 mg/ml) by about 3.0–4.0 folds (Table 4). According to this observation, encapsulation with GG-gum improved the GCSP-antibacterial activity. This improvement may be due to a much increase in the antioxidant activity of the S/GG-microcapsule (141.6%). Some antioxidant-polyphenols induced oxidative injury to bacteria by releasing H_2_O_2_, causing damage to the bacterial cell membrane and/or inhibiting bacterial growth (Matejczyk et al., 2018). In addition, the reduced particle sizes of the prepared S/GG-microencapsulate mainly help in faster cellular uptake causing interference with protein synthesis and more damage to the bacteria cell membrane (Nakayama et al., 2012). The improved antibacterial activity of some encapsulates was previously reported such as CEO-encapsulate and CS/GTO-encapsulate demonstrated enhanced antibacterial activity against *S. aureus* and *E. coli* by 1.16, 9.4 and 1.28, 4.7 folds, respectively, compared to their non-encapsulated forms (Esmaeili and Asgari, 2015; Shetta et al., 2019).

**Table 4:** Antibacterial efficacy evaluated as minimum bactericidal concentration (MBC) for GCSP and the prepared S/GG-microencapsulate. Amoxicillin is used as a positive control

Sample	Pathogenic bacterial strain MBC (mg/ml)			
	*S. aureus*	*B. subtilis*	*E. coli*	*S. typhi*
GCSP	3.0 ± 0.11^a^	3.5 ± 0.08^a^	2.7 ± 0.10^a^	2.9 ± 0.41^a^
S/GG-encapsulate	1.0 ± 0.01^b^	1.3 ± 0.02^b^	0.7 ± 0.01^b^	0.91 ± 0.01^b^
Amoxicillin	1.2 ± 0.05^c^	2.5 ± 0.13^c^	2.3 ± 0.31^c^	ND

In conclusion, this study is the first report on the encapsulation of upgraded-GCSP using the freeze-drying technique. Coating materials M and GG-gum and their mixtures at different ratios besides the sonication technology showed a great influence on the encapsulation of the GCSP. Among all the prepared microcapsules for GCSP, the S/GG-microcapsule retained the highest phenolic content, antioxidant activity, and encapsulation efficiency. The S/GG-microcapsule also showed a greater release of GCS-phenolic compounds in the simulated intestinal fluid than in gastric fluid implying that GG-gum protects the phenolic compounds from gastric degradation and improves their bio-accessibility in the intestine. S/GG was suitable for long-term storage at 40 °C and possessed better physical properties, including moisture, solubility, swelling, and morphological structures than M and S/M-microcapsules. Additionally, the FTIR-spectra of the S/GG-microcapsule confirmed the association of the GG-gum and GCSP functional groups. Moreover, enhanced thermal stability was demonstrated for S/GG-microcapsule that reached 400 °C. Finally, the S/GG-microcapsule exhibited significantly improved antibacterial activity toward all the tested bacteria. Overall, the GG-gum is an effective cover for the conservation of the phenolic compounds of the GCSP, enhancing their antioxidant and antibacterial activities, extending their shelf life, and improving their thermal stability. Hence, this microcapsule can be added to different food models to utilize its therapeutic/biological benefits as well as can be used as a potent antibacterial agent.
